# Evaluation of the Effectiveness of TECAR and Vibration Therapy as Methods Supporting Muscle Recovery After Strenuous Eccentric Exercise

**DOI:** 10.3390/jcm14186648

**Published:** 2025-09-21

**Authors:** Łukasz Oleksy, Anna Mika, Maciej Daszkiewicz, Martyna Sopa, Miłosz Szczudło, Maciej Kuchciak, Artur Stolarczyk, Olga Adamska, Paweł Reichert, Zofia Dzięcioł-Anikiej, Renata Kielnar

**Affiliations:** 1 Department of Orthopedics, Traumatology and Hand Surgery, Faculty of Medicine, Wroclaw Medical University, 50-556 Wroclaw, Poland; loleksy@oleksy-fizjoterapia.pl (Ł.O.); pawel.reichert@umw.edu.pl (P.R.); 2 Oleksy Medical & Sport Sciences, 37-100 Łańcut, Poland; 3 Institute of Clinical Rehabilitation, University of Physical Culture in Kraków, 31-571 Kraków, Poland; 4 Physiotherapy Research Laboratory, University Centre of Physiotherapy and Rehabilitation, Faculty of Physiotherapy, Wroclaw Medical University, 50368 Wroclaw, Poland; maciej.daszkiewicz@student.umw.edu.pl; 5 Institute of Applied Mechanics, Faculty of Mechanical Engineering, Poznan University of Technology, 61-138 Poznań, Poland; martyna.sopa@put.poznan.pl; 6 Centre of Sport and Recreation, University of Rzeszów, 35-959 Rzeszów, Poland; mszczudlo@ur.edu.pl; 7 Department of Physical Education, University of Rzeszow, 35-959 Rzeszów, Poland; mkuchciak@ur.edu.pl; 8 Department of Orthopaedics and Rehabilitation, Medical and Dentistry Faculty, Medical University of Warsaw, 02-091 Warsaw, Poland; artur.stolarczyk@wum.edu.pl; 9 Department of Ophthalmology, Faculty of Medicine, Collegium Medicum Cardinal Stefan Wyszyński University in Warsaw, 01-815 Warsaw, Poland; o.adamska@uksw.edu.pl; 10 Department of Rehabilitation, Medical University of Bialystok, 24A M. Skłodowskiej-Curie St., 15-276 Bialystok, Poland; zofia.dzieciol-anikiej@umb.edu.pl; 11 Faculty of Health Sciences and Psychology, Collegium Medicum, Institute of Physiotherapy, University of Rzeszów, 35-315 Rzeszów, Poland; kielnarrenata@o2.pl

**Keywords:** TECAR, vibration therapy, eccentric exercise, muscle fatigue, sport, injury, recovery

## Abstract

**Background/Objectives**. Despite growing interest in capacitive-resistive electric transfer TECAR) and Vibration therapy (VT), their comparative effectiveness in sports recovery remains unclear. This study aimed to evaluate and contrast the short-term effects of TECAR and VT on neuromuscular recovery following eccentric muscle fatigue, relative to passive rest, in active young adults. We hypothesized that both interventions would accelerate recovery and potentially reduce injury risk. **Methods**. Forty-one participants were randomized into two groups: TECAR therapy (Group 1) and VT (Group 2). Neuromuscular function was assessed at baseline, post-exercise, and post-intervention using tensiomyography (TMG) and electromyography (EMG). **Results**. Both groups showed a significant increase in EMG MDF intercept after exercise. Post-intervention, VT induced a further rise in this parameter, whereas TECAR stabilized values without significant change. In the contralateral resting limb, increases persisted after exercise and passive recovery. Between-limb differences were significant only in the TECAR group. TMG analysis revealed a non-significant but large-effect increase in contraction delay (Td) post-exercise, followed by significant reductions after both interventions. In the left limb, Td changes were not significant. For maximal displacement (Dm), both VMO and VLO muscles demonstrated a significant decrease post-exercise and a marked recovery after both therapies. Other TMG parameters (Ts, Tc, Tr) showed no significant changes. **Conclusions**. Both TECAR and VT effectively enhanced neuromuscular recovery after eccentric exercise. TECAR demonstrated a modest but consistent advantage, particularly in normalizing muscle recruitment and restoring mechanical properties, making it suitable in contexts requiring rapid recovery. VT, however, remains a more accessible and cost-effective modality. These findings support the application of both techniques in sports recovery, while highlighting the need for further research in professional athletes and diverse exercise settings to optimize regeneration strategies and reduce injury risk.

## 1. Introduction

Eccentric muscle contractions, commonly observed in sports, are strongly associated with a high risk of exercise-induced muscle damage (EIMD) [1,2]. Repeated deceleration, landing, or direction-changing movements impose significant mechanical stress on muscle fibers, leading to microtrauma and triggering a cascade of inflammatory responses, along with alterations in peripheral neural input [3]. A hallmark symptom of EIMD is delayed-onset muscle soreness (DOMS), which typically emerges within 24 h post-exercise. DOMS manifests as pain during movement or palpation, a reduction in maximal voluntary muscle contraction force, decreased joint range of motion, diminished stretch reflex sensitivity, and impaired postural control [1,4].

Minimizing fatigue and accelerating recovery are essential components of optimizing athletic performance, particularly in disciplines with high training volumes and intense physical demands. In parallel, recovery strategies have garnered increased attention among athletes, coaches, and sports medicine practitioners, aiming to reduce fatigue-related performance decline [5,6]. Traditional modalities such as massage, hydrotherapy, stretching, active recovery, compression garments, and cold-water immersion have shown inconsistent results in the literature, with some studies reporting physiological benefits [7,8] and others indicating minimal or no effect [9,10]. These inconsistencies may depend on factors such as the timing of the intervention relative to exercise, stimulus intensity, participants’ training status, and individual variability in biological responses—variables that are often underreported. Among the emerging approaches, physical therapies based on electromagnetic or mechanical stimulation—such as capacitive-resistive electric transfer (TECAR) and vibration therapy (VT)—are gaining momentum due to their potential to target deep tissue structures and support biological recovery processes [5,11]. To accurately assess the benefits of TECAR and VT, it is important to consider these moderating factors, which may determine the effectiveness of recovery interventions and clarify their potential advantage over traditional methods.

TECAR therapy is a non-invasive form of endogenous thermotherapy that utilizes high-frequency electromagnetic currents—typically in the range of 300 kHz to 1 MHz—to stimulate tissue regeneration and accelerate post-exercise recovery [5,12,13]. Preclinical studies suggest that the heating effect of TECAR therapy promotes vasodilation, enhances microcirculation, and increases hemoglobin saturation, thereby facilitating oxygen delivery, nutrient transport, and metabolic waste removal. These processes contribute to reduced muscle stiffness, alleviated fatigue, improved muscle flexibility, and accelerated reabsorption of edema or hematomas [12,13]. However, clinical confirmation of these effects in controlled studies remains limited, and the extent to which these mechanisms translate into measurable recovery outcomes in humans is not fully established.

Similarly, vibration therapy, particularly in its localized form, has been proposed as a modality for post-exercise recovery, neuromuscular stimulation, and mitigation of exercise-induced muscle damage (EIMD). Physiological studies indicate that VT may enhance local blood flow, improve oxygen resaturation, and accelerate removal of metabolic byproducts such as lactate and hydrogen ions [1,14,15]. These mechanisms are hypothesized to reduce inflammation, diminish pain signaling, and support tissue repair. VT has also been associated with improvements in maximum voluntary contraction and reduced neuromuscular fatigue, likely via enhanced motor unit recruitment and central nervous system engagement. Nevertheless, many of these findings stem from studies with limited sample sizes or preclinical models, and robust clinical evidence confirming these effects is still sparse [16,17].

Consequently, there is a growing interest in investigating the efficacy of physical therapies such as VT and TECAR in supporting post-exercise muscle recovery, particularly in the context of professional and recreational sports. However, comparative data evaluating the effectiveness of VT relative to other physiotherapeutic approaches remain limited, highlighting the need for well-controlled studies to distinguish between theoretical mechanisms and clinically verified outcomes.

Although TECAR and VT have gained popularity in recent years, few studies have directly compared their effectiveness in sports and rehabilitation settings. Therefore, the aim of this study was to examine and contrast the short-term effects of TECAR and VT on recovery following eccentric muscle fatigue, with particular emphasis on neuromuscular function. Specifically, we sought to determine whether either intervention provides superior recovery benefits compared to passive rest when applied shortly after an exhaustive training session in active young adults. We further hypothesized that by supporting post-exercise recovery, these methods may help reduce tissue overload and thereby potentially contribute to lowering the risk of injuries during sports activities.

## 2. Materials and Methods

### 2.1. Study Participants

Forty-one healthy and physically active adult participants were enrolled in this study (Table 1). Inclusion criteria required participants to be aged 18–25 years, free from lower limb pain, injuries, or diseases within the past year, and without any systemic illnesses. Participants were recruited via university social media channels. Following enrollment, participants were randomly assigned according to the order of arrival to begin with TECAR or VT recovery.

Group 1 (*n* = 20)–VT recovery
Group 2 (*n* = 21)–TECAR recovery

This randomization ensured that each participant had an equal chance of being allocated to either intervention, while maintaining balanced group sizes.

Participants were fully informed about the research protocol and provided their written informed consent to participate in the study. They were familiarized with the study procedures prior to the measurements. Ethical approval from the Ethical Committee at the Regional Medical Chamber in Kraków (35/KBL/OIL/2024) was obtained. All procedures adhered to the principles of the 1964 Declaration of Helsinki and its later amendments.

### 2.2. Study Design

All measurements were conducted during the morning hours by the same researchers, who were well-trained and experienced with both the equipment and the test protocol. Body mass (kg) and height (cm) were recorded prior to testing. All participants performed a 10 min warm-up before the tests. Both limbs were evaluated. The right limb was the experimental one (receiving TECAR or VT recovery), and the left limb served as a control with passive rest only.

Assessments were performed at three time points: at baseline, after the fatigue protocol, and following the recovery intervention. At each of these time points, participants were assessed for tensiomyography (TMG) and electromyography (EMG).

### 2.3. Procedures

#### 2.3.1. EMG Measurements

EMG activity of the right and left vastus lateralis oblique (VLO) muscles was recorded in accordance with the Surface Electromyography for the Non-Invasive Assessment of Muscles (SENIAM) guidelines [18,19]. Prior to electrode placement, the skin was cleansed with alcohol, and EMG sensors were positioned along the longitudinal axis of the VLO muscle bellies. The signals were collected using Delsys Trigno sensors (Delsys, Natick, MA, USA). Surface EMG activity of the target muscles was measured during a 60 s isometric contraction, performed in a standing half-squat position with the back supported against a wall [20]. We selected this specific type of test exercise because, according to established EMG measurement methodology and signal analysis principles, the most reliable and stable assessment of frequency changes is achieved during submaximal isometric contractions. Such conditions minimize the confounding effects of uncontrolled movements of the muscle belly, which are known to impair the quality and reproducibility of EMG recordings [21,22,23]. This approach was therefore adopted to ensure greater validity and consistency of the recorded signal.

The EMG data were collected at a sampling frequency of 1500 Hz and processed according to SENIAM guidelines [18,19,24]. The signal was processed with MATLAB 4.0 software (The MathWorks, Natick, MA, USA). To prepare the data for further analysis, the signal was processed using a 4th-order Butterworth bandpass filter with cutoff frequencies ranging from 20 Hz to 500 Hz. The 4th-order Butterworth filter was applied bidirectionally to prevent phase shift. Additionally, EMG signals were visually inspected for movement artifacts, and any contaminated segments were excluded prior to analysis. Subsequently, the 60 s signals were segmented into 0.5 s windows and processed using the Continuous Wavelet Transform (CWT) tool, employing the Daubechies db4 wavelet family with 128 scales. The median frequency (MDF) was extracted from each 0.5 s window for further analysis. The Daubechies db4 wavelet was chosen for the continuous wavelet transform (CWT) based on prior studies [24,25] indicating its suitability for EMG signals, providing a reliable balance between time and frequency resolution for median frequency (MDF) analysis.

Three parameters were computed to evaluate muscle performance during the isometric task: MDF, slope, and intercept [24,25]. MDF represents the frequency below which 50% of the EMG signal’s power is contained and is commonly used as an indicator of muscle fatigue, as a shift toward lower frequencies typically occurs with prolonged muscle activity. The slope and intercept are derived from the linear regression of MDF over time: the slope quantifies the rate of change in frequency (i.e., the progression of fatigue), while the intercept represents the initial frequency at the start of the measurement. These parameters were chosen because they provide a straightforward and widely accepted method to quantify both the onset and progression of muscle fatigue, allowing us to evaluate changes in muscle function over the course of the task. Recent studies have reported ICC values for changes in median frequency (MDF) ranging from 0.70 [26] to 0.99 [27], indicating moderate to excellent repeatability. The full EMG signal processing procedure has been described in our previous study [28].

#### 2.3.2. TMG Measurements

Tensiomyography was used to assess muscle function by measuring the following variables: muscle displacement (Dm), contraction time (Tc), delay time (Td), sustain time (Ts), and relaxation time (Tr). ICC values for TMG parameters ranged from 0.75 to 0.99, with coefficient of variation (CV) values between 2% and 8%, reflecting good to excellent repeatability [29]. Measurements were obtained with a digital displacement transducer (GK 40, Panoptik d.o.o., Ljubljana, Slovenia) equipped with a spring stiffness of 0.17 N/mm. The sensor was positioned perpendicular to the muscle belly with an initial pressure of 1.5 × 10^−2^ N/mm^2^, placed on the thickest part of the muscle identified visually and by palpation during voluntary contraction. Assessments were conducted on the vastus lateralis oblique (VLO) and vastus medialis oblique (VMO) muscles of both limbs. Muscle stimulation was delivered using two self-adhesive electrodes (Axelgaard, Pulse, Fallbrook, CA, USA) placed 2–5 cm apart and connected to an electrostimulator (TMG-S1, Furlan and Co., Ltd., Ljubljana, Slovenia) producing a 1 ms pulse. Stimulation began at 30 mA and was increased in 10 mA increments up to a maximum of 100 mA. The maximum stimulation amplitude was defined as the lowest current capable of producing the maximum muscle displacement [29].

#### 2.3.3. Fatiguing Eccentric Protocol

Participants stepped onto a 20 cm box, followed by a 40 cm box, and then performed drop landings at a rate of 30 repetitions per minute. Each protocol consisted of five sets with 1 min rest intervals [30].

#### 2.3.4. VT Intervention

The VT intervention was conducted with a pneumatic vibrator Vibra 3.0 (Vibra Plus, A Circle s.p.a., San Pietro in Casale, Bologna, Italy) powered by compressed air vibrations, operating in the frequency range of 10–300 Hz. A frequency of 50 Hz was applied to the right quadriceps femoris muscle in sitting position using a cup-shaped transducer with a 5 cm contact surface for 20 min. This frequency was selected based on other authors’ findings, who reported significant improvements in muscle strength and endurance following low-frequency vibration massage (1–50 Hz). The 50 Hz frequency is within the effective range for neuromuscular stimulation, promoting muscle relaxation and enhancing recovery post-exercise [31]. Additionally, it was demonstrated that low-frequency whole-body vibration (2–30 Hz) effectively reduces muscle fatigue and enhances recovery in untrained individuals [32,33]. The 20 min duration and the use of a cup-shaped transducer with a 5 cm contact surface were selected to ensure consistent and sufficient stimulation of the targeted muscle while maintaining participant comfort

#### 2.3.5. TECAR Intervention

The TECAR intervention was administered to the right quadriceps femoris muscle using the BACK4 device (Winback Europa SAS, Biarritz, France) in capacitive (CAP) mode for a duration of 20 min. The therapy was applied with the signal at a variable power with a maximum of 300 W. The frequency used was 500 kHz with an intensity of 40%. During the procedure, participants were seated, and a metallic plaque was positioned under the thigh. The intervention was standardized to a distal-to-proximal stroking rhythm with moderate pressure [13,34,35]. For the TECAR intervention, the capacitive (CAP) mode at 500 kHz with 40% intensity and a maximum power of 300 W was chosen to optimize deep tissue heating and promote local blood flow and tissue recovery. The distal-to-proximal stroking rhythm with moderate pressure was standardized to ensure reproducibility and to facilitate even energy distribution throughout the muscle. The 20 min duration reflects protocols commonly used in therapeutic settings for muscle recovery and rehabilitation [36].

### 2.4. Statistical Analysis

Statistical analyses were performed using the STATISTICA 13.0 software package. Data distribution was assessed with the Shapiro–Wilk test and was found to be normal. The ANOVA with repeated measurement was performed (group × time) to determine the differences in EMG and TMG variables between groups and between three measurements. The *t*-test was used to evaluate the differences between right (experimental) and left (control) limbs. Differences were considered statistically significant if the level of test probability was lower than the assumed level of significance (*p* < 0.05). The effect size was calculated using Cohen’s d and interpreted as small (to 0.1), medium (0.4), or large (>0.8) [37]. Power analysis indicated that at least 30 subjects were required to obtain a power of 0.8 at an alpha level = 0.05 with an effect size of d = 0.8 and a minimum ICC of 0.50.

## 3. Results

### 3.1. Electromyography (EMG)

**Figure 1:**
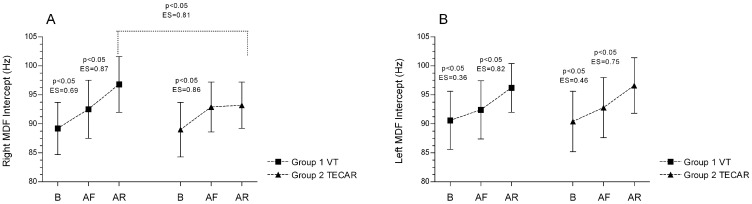
Differences in MDF intercepts between groups and across three measurements for the Right (**A**) and Left (**B**) sides. Values are expressed as mean ± SD; *p*—*p*-value; ES—effect size; MDF—median frequency, B-baseline; AF—after fatigue; AR—after recovery.

Following eccentric fatigue, both groups demonstrated a significant increase in the MDF intercept. In Group 1, which underwent vibration therapy (VT) recovery, this EMG parameter exhibited a further significant rise. In contrast, in Group 2, which received TECAR recovery, post-recovery measurements of bioelectrical activity did not indicate any additional increase in the intercept; the observed change was minimal compared to post-exercise values and did not reach statistical significance (Figure 1A). In the contralateral (left) limb, which served as a control and was subjected to passive rest, both groups showed a significant increase in the intercept after exercise and after passive recovery (Figure 1B). A significant between-limb difference was observed exclusively in Group 2, following the TECAR recovery intervention (R vs. L; 93.2 ± 4.0 Hz vs. 96.6 ± 4.8 Hz; *p* < 0.05; ES (large) = 0.79). For the remaining EMG parameters (MDF slope and MDF value), non-significant differences were detected—neither between groups, nor between limbs, nor across consecutive measurements (*p* > 0.05).

### 3.2. Tensiomyography (TMG)

**Figure 2:**
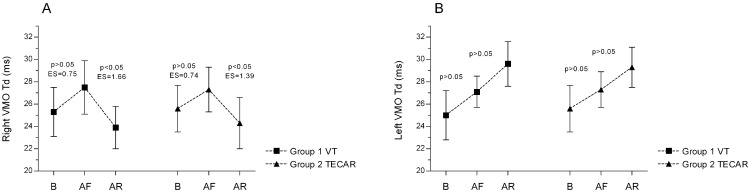
Differences in VMO muscle delay time (Td) between groups and across three measurements for the Right (**A**) and Left (**B**) sides. Values are expressed as mean ± SD; *p*—*p*-value; ES—effect size; VMO—vastus medialis oblique muscle, B-baseline; AF—after fatigue; AR—after recovery.

In our study, the VMO muscle in both groups demonstrated an increase in Td following exercise (Figure 2A). Although this change did not reach statistical significance, it was accompanied by a high effect size. After recovery, however, both groups exhibited a significant reduction in Td, indicating a beneficial effect of both vibration therapy (VT) and TECAR recovery (Figure 2A). In the left limb, Td increased after exercise and after recovery in both groups, but these changes were not significant (Figure 2B). Similarly, in the VLO muscle, no significant differences were observed—neither between successive measurements, nor between groups, nor between limbs (*p* > 0.05).

**Figure 3:**
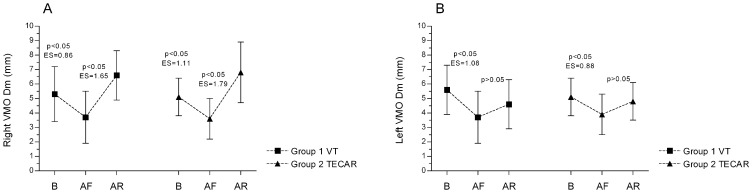
Differences in VMO muscle displacement (Dm) between groups and across three measurements for the Right (**A**) and Left (**B**) sides. Values are expressed as mean ± SD; *p*—*p*-value; ES—effect size; VMO—vastus medialis oblique muscle, B—baseline; AF—after fatigue; AR—after recovery.

**Figure 4:**
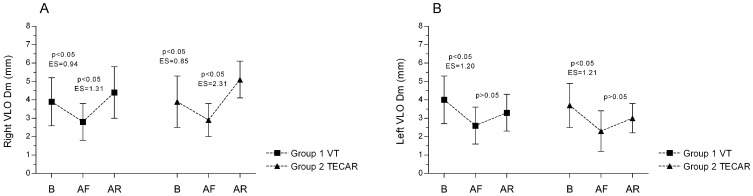
Differences in VLO muscle displacement (Dm) between groups and across three measurements for the Right (**A**) and Left (**B**) sides. Values are expressed as mean ± SD; *p*—*p*-value; ES—effect size; VLO—vastus lateralis oblique muscle, B-baseline; AF—after fatigue; AR—after recovery.

For the Dm parameter, both the VMO and VLO muscles exhibited a similar pattern of changes following exercise and subsequent recovery. In the right limb, exercise induced a significant decrease in Dm, which was then followed by a substantial and significant increase after both recovery interventions, with a large effect size (Figure 3A and Figure 4A). In the left limb, Dm also decreased significantly after exercise; however, after 20 min of passive rest, only a non-significant upward trend was observed (Figure 3B and Figure 4B). Between-limb comparisons revealed significant differences in both groups in the post-recovery measurements (Group 1: R vs. L; VMO: 6.6 ± 1.7 mm vs. 4.6 ± 1.7 mm, *p* < 0.05, ES (large) = 1.17; VLO: 4.4 ± 1.4 mm vs. 3.3 ± 1.0 mm, *p* < 0.05, ES (large) = 0.90), (Group 2: R vs. L, VMO: 6.8 ± 2.1 mm vs. 4.8 ± 1.3 mm; *p* < 0.05, ES (large) = 1.14; VLO: 5.1 ± 1.0 mm vs. 3.0 ± 0.8 mm, *p* < 0.05, ES (large) = 2.31). No significant differences were found for the remaining TMG parameters (Ts, Tc, Tr) (*p* > 0.05).

## 4. Discussion

The aim of the present study was to evaluate the effectiveness of two methods supporting recovery after eccentric exercise—TECAR therapy and vibration therapy (VT)—in young, recreationally active individuals. The results indicated that both methods significantly improved the assessed neuromuscular parameters, which is consistent with previous reports confirming the efficacy of both deep radiofrequency thermotherapy and vibratory stimulation in reducing post-exercise muscle fatigue symptoms and enhancing motor function [5,11,16]. These findings align with earlier evidence highlighting the importance of appropriately selected recovery methods in improving muscle function following exercise [7,8,16]. Commonly used recovery techniques, such as massage, compression garments, or cryotherapy, have yielded inconsistent outcomes [7,8,9,10]. Consequently, there is growing interest in alternative, targeted approaches such as deep radiofrequency thermotherapy and vibration therapy, which—as demonstrated by the present results—may serve as effective tools to support post-exercise recovery.

The mechanisms of action of the two methods are different, although the final outcome—improvement in functional parameters—remains similar. In the case of TECAR therapy, the observed effects are associated with the influence of radiofrequency waves in the range of 300 kHz–1 MHz on body tissues, leading to a controlled increase in temperature in both superficial and deep layers [5,11]. This thermal process improves microcirculation, enhances hemoglobin saturation, and accelerates the removal of metabolic by-products, thereby promoting post-exercise recovery [11,38]. These effects may explain the faster recovery of muscle function parameters observed in the present study, which is consistent with the findings of Duñabeitia et al. [5], who reported that TECAR following eccentric exercise resulted in greater stride length and improved step angle in runners compared to passive rest. Further evidence also indicates that TECAR may be more effective than ultrasound therapy in enhancing hamstring flexibility [39]. However, the authors indicated that when TECAR was combined with static stretching, the outcomes were comparable to static stretching alone, suggesting that its efficacy may depend on the context and method of application [39]. Ribeiro et al. [40] reported that the group that received a TECAR intervention after exercise experienced lower pain levels compared to the control group. Moreover, in the Single Hop Test, both groups demonstrated significant increases in jump length, but the control group showed a smaller increase in strength than the TECAR group [40].

Additionally, previous studies using TMG as an evaluation method have shown that TECAR may positively influence muscle stiffness and contraction delay time, suggesting improvements in the elastic properties of muscle fibers and the optimization of their performance after exercise [5,41,42]. Our findings confirm these observations, as these parameters improved in both groups.

Vibration therapy (VT), particularly in its local form, primarily acts by increasing blood flow in muscles and modulating muscle spindle excitability, which can elevate the pain threshold and improve synchronization of motor units [1,16]. Support for the analgesic effects of VT on pain perception, especially in the context of muscle soreness, has been provided by several studies on post-exercise recovery modalities [2,16,17]. Cochrane et al. [16] demonstrated that VT reduced peak muscle soreness by 34% following eccentric exercise, while Manimmanakorn et al. [43] suggested that this mechanism may be comparable to active recovery, enhancing lactate and H^+^ ion clearance. In the present study, the VT group demonstrated improvements in TMG parameters and partial recovery in EMG indicators, indicating a positive effect on muscle function. However, the increase in MDF intercept values after recovery suggests that the return to full recruitment balance of motor units was slower compared to TECAR therapy. It is important to note that the effectiveness of VT depends on the specific protocol used, including frequency, application time, intermittent versus continuous modes, and muscle load during treatment. Local muscle vibration applied to relaxed muscles may increase blood flow more effectively than whole-body vibration, where the mechanical stimulus is partially dispersed into adjacent tissues [44]. These results highlight the importance of VT not only as a therapeutic method with potential clinical applications, but also as a supportive approach for optimizing recovery in sports and, by accelerating post-exercise recovery, as a means of preventing musculoskeletal injuries in athletes. The intercept, defined as the point of intersection of the regression line with the y-axis, provides valuable insight into the spectral behavior of the EMG signal during muscle fatigue. Previous studies have consistently shown that fatigue is associated with a shift in signal energy toward lower frequencies, reflected in a decline in median frequency (MDF), alterations in waveform dynamics, and increased energy distribution at higher scales corresponding to lower frequencies [27,45]. These changes are often accompanied by a progressive increase in the intercept value, which can therefore be interpreted as an indirect marker of neuromuscular fatigue [45,46]. Our findings align with this established pattern in the VT recovery group, where an increase in the intercept was evident. This observation indicates a shift in EMG spectral content toward lower frequencies and corresponds with the expected manifestation of muscle fatigue, consistent with earlier reports [44,47]. In contrast, no significant increase in intercept was detected following TECAR recovery, suggesting that TECAR may have mitigated or delayed the spectral shift typically associated with fatigue, potentially by enhancing microcirculation and promoting more efficient muscle fiber recruitment. The absence of intercept elevation following TECAR further reinforces its potential role in accelerating recovery and sustaining neuromuscular performance. The results of our study suggest that while both methods are effective in improving muscle function, TECAR may act more rapidly and comprehensively at the levels of microcirculation and muscle fiber regeneration. This effect may be attributed to the synergy of its thermal and non-thermal mechanisms, which influence both cellular metabolism and the mechanical properties of tissues [5,11,38]. It should be emphasized, however, that the advantage of TECAR over VT in our study was moderate and may be particularly relevant in situations requiring the fastest possible return to full functional capacity—for instance, during athletes’ competition preparation. On the other hand, VT remains a more accessible method, less dependent on specialized equipment, while still providing significant regenerative benefits, as confirmed by earlier studies [1,44].

In the present study, the use of TMG and EMG allowed for a precise assessment of changes in muscle excitability. Tensiomyography revealed alterations in two of the five evaluated parameters: delay time and maximal displacement. Delay time represents the period between the electrical stimulus and the attainment of 10% of maximal contraction. An increase in Td reflects a slower muscular response to stimulation and may indicate fatigue, reduced neuromuscular excitability, or deficits in activation. Conversely, a decrease in Td signifies a faster response, suggesting improved neuromuscular conduction. Typically, exercise-induced fatigue is associated with an increase in Td, reflecting delayed muscle responsiveness, whereas recovery may lead to a reduction in Td, consistent with enhanced neuromuscular function [48,49]. In our study, both groups exhibited an increase in delay time following exercise. Although this change did not reach statistical significance, it was accompanied by a high effect size. After recovery, however, both groups showed a significant decrease in Td, confirming the positive effects of both vibration and TECAR recovery. In the left limb, Td increased after both exercise and recovery, but these changes were not significant. The second parameter that demonstrated sensitive and reliable changes was maximal displacement, which reflects the amplitude of muscle belly movement during contraction and is commonly interpreted as an indicator of muscle stiffness or softness. An increase in Dm suggests greater compliance and reduced resting tension, whereas a decrease indicates higher stiffness and greater passive tension, often resulting from fatigue, injury, or compensatory contraction. Fatigue is typically associated with a reduction in Dm, reflecting stiffer, less elastic muscle tissue, while recovery interventions tend to restore compliance and increase Dm [48,49]. In the present study, exercise induced a significant decrease in Dm, followed by a substantial and statistically significant increase after both recovery interventions. The use of TMG and EMG in our study enabled a precise evaluation of the effectiveness of different interventions aimed at enhancing muscle function following fatigue. These indicators may serve as sensitive tools for assessing the level of post-exercise muscle recovery and for monitoring the extent of muscle fatigue during training. Such applications could facilitate the early implementation of recovery strategies, thereby reducing the risk of sports-related injuries.

The limitations of this study include the relatively small sample size and the absence of a crossover design, which restricts the generalizability of the findings and the ability to draw firm conclusions regarding the superiority of one method over the other. Moreover, no direct measurements of physiological indicators such as lactate concentration were performed, which could have complemented the interpretation of the mechanisms underlying the effects of both approaches. Future research should involve larger participant groups, various loading protocols, and comparisons of the effectiveness of TECAR and VT in professional athlete populations, with an analysis of long-term outcomes. It is also worth considering the use of simultaneous EMG and TMG monitoring to more precisely track changes in motor unit recruitment and the mechanical properties of muscles during the recovery process.

## 5. Conclusions

This study compared TECAR and VT in the context of recovery following eccentric exercise in young, active individuals. Both modalities improved neuromuscular parameters, consistent with previous evidence on the effects of deep thermotherapy and local vibration. TECAR therapy showed a trend toward faster normalization of neuromuscular activity and mechanical muscle properties, although the small sample size and study design limit definitive conclusions about its superiority over VT. Both interventions can be considered effective recovery tools, with VT offering greater portability and accessibility. Future large-scale studies involving professional athletes and diverse exercise protocols are needed to confirm these preliminary observations and further inform recovery strategies in sport.

## Figures and Tables

**Table 1:** Subjects’ characteristics.

	Group 1	Group 2
Number of subjects (*n*)	20	21
Male/Female	3/17	4/17
Height (cm)	168 ± 10	167 ± 7
Weight (kg)	63 ± 14	64 ± 12
Age	19 ± 2	19 ± 2

## Data Availability

All data generated or analyzed during this study are included in this published article.
